# Excess Cardiovascular Risk Burden in Jamaican Women Does Not Influence Predicted 10-Year CVD Risk Profiles of Jamaica Adults: An Analysis of the 2007/08 Jamaica Health and Lifestyle Survey

**DOI:** 10.1371/journal.pone.0066625

**Published:** 2013-06-21

**Authors:** Marshall K. Tulloch-Reid, Novie O. Younger, Trevor S. Ferguson, Damian K. Francis, Abdullahi O. Abdulkadri, Georgiana M. Gordon-Strachan, Shelly R. McFarlane, Colette A. Cunningham-Myrie, Rainford J. Wilks, Simon G. Anderson

**Affiliations:** 1 Tropical Medicine Research Institute, The University of the West Indies, Mona, Kingston, Jamaica; 2 Department of Economics, The University of the West Indies, Mona, Kingston, Jamaica; 3 Dean’s Office, Faculty of Medical Sciences, The University of the West Indies, Mona, Kingston, Jamaica; 4 Department of Community Health and Psychiatry, The University of the West Indies, Mona, Kingston, Jamaica; 5 Institute of Cardiovascular Sciences, University of Manchester, Manchester, United Kingdom; Fundación para la Prevención y el Control de las Enfermedades Crónicas No Transmisibles en América Latina (FunPRECAL), Argentina

## Abstract

**Background:**

Black Caribbean women have a higher burden of cardiovascular disease (CVD) risk factors than their male counterparts. Whether this results in a difference in incident cardiovascular events is unknown. The aim of this study was to estimate the 10 year World Health Organization/International Society for Hypertension (WHO/ISH) CVD risk score for Jamaica and explore the effect of sex as well as obesity, physical activity and socioeconomic status on these estimates.

**Methods and Findings:**

Data from 40–74 year old participants in the 2007/08 Jamaica Health and Lifestyle Survey were used. Trained interviewers administered questionnaires and measured anthropometrics, blood pressure, fasting glucose and cholesterol. Education and occupation were used to assess socioeconomic status. The Americas B tables were used to estimate the WHO/ISH 10 year CVD risk scores for the population. Weighted prevalence estimates were calculated. Data from 1,432 (450 men, 982 women) participants were analysed, after excluding those with self-reported heart attack and stroke. The women had a higher prevalence of diabetes (19%W;12%M), hypertension (49%W;47%M), hypercholesterolemia (25%W;11%M), obesity (46%W;15%M) and physical inactivity (59%W;29%M). More men smoked (6%W;31%M). There was good agreement between the 10-year cardiovascular risk estimates whether or not cholesterol measurements were utilized for calculation (kappa –0.61). While 90% had a 10 year WHO/ISH CVD risk of less than 10%, approximately 2% of the population or 14,000 persons had a 10 year WHO/ISH CVD risk of ≥30%. As expected CVD risk increased with age but there was no sex difference in CVD risk distribution despite women having a greater risk factor burden. Women with low socioeconomic status had the most adverse CVD risk profile.

**Conclusion:**

Despite women having a higher prevalence of CVD risk factors there was no sex difference in 10-year WHO/ISH CVD risk in Jamaican adults.

## Introduction

Cardiovascular Diseases are the leading cause of death globally. In 2008 an estimated 17.1 million people died from cardiovascular disease (CVD), representing 29% of all global deaths [1]. Cardiovascular diseases are also the leading cause of death in low and middle income countries [2], [3]. In addition disproportionately more CVD deaths occur at a younger age in low and middle income countries than in developed countries resulting in substantial loss of productive years. [2], [4].

In Jamaica, CVD is now the leading cause of death with an estimated crude mortality rate from ischemic heart disease and stroke of 110 per 100 000 in 2008 [5]. This is consistent with local national surveys which have demonstrated a high prevalence of CVD risk factors such as obesity, hypertension, diabetes and elevated cholesterol in Jamaica [6]. The 2007/08 Jamaica Lifestyle Survey II found that among 15–74 year olds, a quarter of the population was obese and 25% had hypertension. Between 8–15% had diabetes, an elevated total cholesterol and were cigarette smokers [6]. With the exception of smoking, CVD risk factors were more common in women [3], [7]–[9]. In Jamaica these sex differences in CVD risk factors are evident in adolescence and young adulthood [10].

Despite having good data on risk factor prevalence, there are limited data on incident CVD in Jamaica, due to the lack of data systems to track incident (fatal and non-fatal) cardiovascular events and limited cohort data to predict the burden from adverse CVD outcomes [9], [11]. As a result it is not possible to assess the impact of the sex difference in CVD risk factors on outcomes. From the Jamaica Health and Lifestyle Survey, among the 15–74 year old participants the self-reported prevalence of stroke and heart attack was less than 1.5% with no significant sex difference [12]. Self-reported prevalence, like mortality statistics, underestimates the burden of CVD and may reflect differences in survival rather than incidence of these conditions.

Cardiovascular risk scores are routinely used to identify patients at high near term risk of a cardiovascular event for more aggressive prevention and intervention efforts as one means of reducing CVD [13]. Prediction scores such as the Framingham Risk Score or Systematic Coronary Risk Evaluation (SCORE) utilize a combination of clinical and laboratory data [14], [15]. However, many developing countries like Jamaica face two challenges with these methods – firstly, the lack of cohort data to validate these scores which were developed and validated in high-income countries among predominantly Caucasian populations, and secondly, limited access to some of the required laboratory tests for their calculation.

The World Health Organization (WHO) and the International Society of Hypertension (ISH) have produced prediction charts to assess the 10 year risk of a cardiovascular event for all regions globally and can provide similar information on CVD risk as the traditional risk score calculators [16]. It is of particular value in low and middle income countries such as Jamaica where cohort data on CVD incidence are limited and resources for cardiovascular risk assessment are not readily available [16]. Region-specific 10 year risk prediction scores are determined using age, sex, blood pressure, the presence or absence of diabetes and smoking - with or without measured total cholesterol.

The aims of this study were to utilize cardiovascular risk factor prevalence data from the 2007/08 Lifestyle Survey to estimate the 10 year risk of CVD among Jamaican adults 40 years and older (without known CVD) using the WHO/ISH prediction charts and examine whether the sex difference in risk factor burden resulted in differences in predicted 10 year risk. In addition we aimed to assess whether predicted 10 year CVD risk estimates are modified by other CVD risk factors, such as obesity, low physical activity and low socio-economic status, not used for the risk score calculation but which may disproportionately affect women.

## Methods

### Ethics Statement

The study protocol was reviewed and approved by the Ethics Committees of the Faculty of Medical Sciences of the University of the West Indies, Mona, Jamaica, and the Jamaica Ministry of Health. Written informed consent was obtained from each participant prior to enrolment.

### Sampling and Data Collection

The Jamaica Health and Lifestyle Survey II was an island-wide cross-sectional study conducted between November 2007 and February 2008, in a randomly selected sample of 2848 men and women aged 15- 74-years, using a multistage cluster sampling design. Details of the study design were published in a full project report [6] and have been included in Appendix S1. Briefly, the primary sampling units (PSU) consisted of enumeration districts (EDs) selected using a probability proportionate to size method. The EDs were selected in consultation with the Statistical Institute of Jamaica. Within each sampling unit, a random household was selected as the starting point and other households systematically selected at intervals (determined by the number of households in the PSU) to recruit 30 participants within each ED. Within each household, one participant was selected using the Kish random selection method [17]. Additional details on the sampling methodology are also included in Appendix S1.

A standardized questionnaire was administered by trained interviewers.

#### Assessment of risk factors for WHO 10 year cardiovascular risk assessment calculation

Sex was based on interviewer observation. All current smokers and those who quit smoking less than one year before the interview were considered current tobacco smokers. Trained interviewers measured blood pressure using a mercury sphygmomanometer. Three measurements were taken at 1-minute intervals, using the right arm, after the participant had been seated for 5 minutes [18]. The mean of the second and third BP measurements was used in the analysis. Participants were classified as having hypertension if their blood pressure measurement was more than 140/90 mmHg [19] or if they reported taking medications for blood pressure control. Fasting blood glucose and total cholesterol were measured after a 10-hour overnight fast using capillary blood samples (Accutrend® GCT Roche Diagnostics GmbH). Measured capillary glucose was converted to the equivalent fasting plasma glucose using the formula “plasma glucose = 0.102+1.066× capillary glucose” as recommended by the guidelines from the European Association for the Study of Diabetes [20]. Participants were then classified as having diabetes if they had a fasting plasma glucose sample of ≥7.0 mmol/L or if they were on medications for diabetes. Participants were classified as having hypercholesterolemia if they had a fasting total cholesterol ≥5.2 mmol/L or if they reported the use of medications for control of their blood cholesterol. The WHO/ISH risk prediction charts for Americas B were used to calculate each participant’s predicted total cardiovascular risk over the next 10 years.

#### Socioeconomic status

There was a high non-response rate to questions on household income so socioeconomic status was based on occupation and education. The Jamaica Standard Occupational Classification 1991 (JSOC-91) was used to group respondents based on their main lifetime occupation and collapsed into the following categories: highly-skilled (Managers/Professionals), skilled (Clerks/Service Workers including office workers/service industry workers/police or security workers, farmers, craftsmen/tradesmen), semi-skilled/unskilled (Machine Operators/manual workers) and unclassified [21]. Persons were also classified according to whether they had primary, secondary/technical/vocational or tertiary education as their highest level of education.

#### Physical activity

Questions on work and leisure time physical activity from a locally developed questionnaire were used to classify persons into the following three physical activity categories: High, Moderate and Low and Inactive.

#### Obesity

Weight was measured using the Tanita H3000 electronic digital scale and height from a portable height measurement rod. Body mass index was calculated from the weight in kilograms divided by the square of the height in metres and obesity status determined from the WHO criteria [22].

### Statistical Analysis

Data were analysed using Stata 10.0 (Stata Corp., College Station, TX).

All data presented here are for participants 40 years or older, as the WHO/ISH risk prediction scores were designed for this age-group. Data are presented as proportions and their 95% confidence intervals (CI) in each WHO risk factor category stratified by sex. Prevalence estimates and 95% CI were weighted to account for sampling design and selection probabilities. Weighting is a statistical technique designed to remove bias from a survey sample and make the results better project the target population. The initial weighting was done *a priori* to take the probability of selection of each participant into account at the level of the PSU and household. Post stratification weighting for age and sex was performed to correct frame error. Additional details on the calculation of the survey weights are provided in the Appendix S1. The 10 year CVD risk estimates were calculated with and without the use of the total cholesterol measurement.

The kappa statistic was used to test for agreement between prediction scores determined after including or excluding measured cholesterol [23]. The kappa statistic presents the extent to which an observed agreement between two or more measurements exceeds that which would be expected by chance alone.

The kappa statistic was calculated using the formula below.
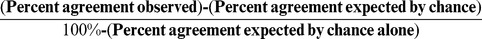



A kappa greater than 0.75 represents excellent agreement while a value of 0.4–0.75 intermediate to good agreement and less than 0.4 poor agreement.

Chi-squared tests, correcting for survey design were used to explore associations between CVD risk and sex for each of the risk factors examined [24]. Chi-squared tests, correcting for survey design were also used to explore associations between CVD risk and demographic, anthropometric and socio-economic characteristics by sex [24]. Absolute numbers were calculated using the percentages and the 2008 population estimates from the Statistical Institute of Jamaica [5].

## Results

Participants, 40 years and older, with self-reported CVD (heart attack or stroke, n = 43) were excluded as the calculator is not used to predict risk in those with established disease. This left a total of 1,432 (450 male, 982 female) participants for the analysis.

Table 1 presents the distribution of the WHO/ISH risk factor categories and cardiovascular risk factors by sex. The prevalence of hypercholesterolemia was 18.4% (16.3–20.8%) with a greater proportion of women than men with elevated cholesterol levels or taking cholesterol medications. Diabetes and hypertension prevalence in those 40 years or older were about 16% and 47% respectively, with women having a higher prevalence of both conditions. Women were more likely to report low levels of physical activity but were less likely to smoke (31% vs 6%). Women also had higher prevalence of obesity (46% vs 15%), and were more likely to have had secondary education or be in the highly skilled occupation group.

**Table 1:** Proportion of population (with 95% CI) in each Cardiovascular Disease Risk Factor category by sex.

Variable	Men(n = 450)	Women(n = 982)	Total (n = 1,432)	P value
**Age (years)+**				
40–49yrs	44.7	45.4	45.0	
50–59yrs	29.2	26.6	27.9	
60–69yrs	18.3	19.4	18.9	
≥70 yrs	7.9	8.7	8.3	
**Cholesterol (mmol/l)** *				
≤4.99	86.6(83.1,89.5)	69.8(66.6,72.7)	78.2(75.8, 80.4)	<0.01
5–5.99	10.2(7.6,13.5)	23.1(20.6,25.8)	16.6(14.8, 18.6)	
6–6.99	2.2(1.2,4.1)	4.7(3.6, 6.1)	3.5(2.7, 4.5)	
7–7.99	1.0(0.3,3.2)	2.5(1.6, 4.0)	1.7(1.1, 2.8)	
**Hypercholesterolemia** *				
No	88.7 (85.7, 91.1)	74.5 (71.1,77.6)	81.6 (79.2, 83.7)	<0.01
Yes	11.3 (8.9, 14.3)	25.5 (22.5, 28.9)	18.4 (16.3, 20.8)	
**Smokes cigarettes** *				
No	68.6(63.9, 72.9)	94.1(92.0, 95.7)	81.4(78.7, 83.8)	<0.01
Yes	31.4(27.1, 36.1)	5.9(4.3, 8.0)	18.6(16.2, 21.3)	
**Diabetes** *				
No	87.3(83.6, 90.3)	80.7(77.9, 83.2)	84.0(81.6, 86.1)	<0.01
Yes	12.7(9.7, 16.4)	19.3(16.8, 22.1)	16.0(13.9, 18.4)	
**Systolic Blood Pressure Category (mmHg)**				
<140	69.3(65.0, 73.3)	70.0(66.1, 73.6)	69.7(66.6, 72.5)	0.91
140–159	19.2(15.6, 23.5)	18.4(15.8, 21.4)	18.8(16.2, 21.8)	
160–179	7.4(5.2,10.3)	6.8(5.0, 9.0)	7.1(5.6, 8.8)	
≥180	4.1(2.1,7.9)	4.8(3.5, 6.5)	4.5(3.2, 6.1)	
**Hypertension** *				
No	55.6 (51.1, 60.6)	50.7 (47.2, 54.2)	53.3 (49.8, 56.7)	0.03
Yes	46.7 (43.3,50.2)	49.3 (45.8, 52.8)	46.7 (43.3, 50.2)	
**BMI (kg/m^2^)** *				
<25.0	50.3 (44.0, 56.5)	21.4 (19.0, 24.1)	35.8 (32.0, 39.8)	<0.01
25–29.9	34.8 (28.9, 41.3)	32.9 (29.9, 36.0)	33.9 (30.4, 37.5)	
≥30.0	14.9 (11.5, 19.0)	45.7 (41.9, 49.5)	30.3 (27.8, 33.0)	
**Physical Activity** *				
High	47.3 (41.2, 53.5)	21.1 (17.9, 24.7)	34.2 (30.5, 38.0)	<0.01
Moderate	23.6 (18.3, 30.0)	19.1 (16.5, 22.0)	21.4 (18.0, 25.1)	
Low	10.0 (7.2, 13.8)	19.1 (16.5, 22.0)	14.6 (12.6, 16.7)	
Inactive	19.1 (15.0, 24.0)	40.8 (36.8, 44.9)	29.9 (26.4, 33.7)	
**Occupational Category**				
Highly Skilled	31.4 (26.4, 36.9)	53.0 (47.1, 58.9)	42.2 (37.8, 46.8)	<0.01
Skilled	52.9 (46.1, 59.5)	11.8 (8.7, 15.9)	32.3 (28.0, 36.9)	
Unskilled	8.4 (5.8, 12.0)	15.7 (12.7, 19.1)	12.0 (9.9, 14.6)	
Unemployed	0	0.6 (0.2, 1.4)	0.3 (0.1, 0.7)	
Other	7.4 (4.8, 11.3)	18.9 (15.3, 23.2)	13.2 (10.8, 15.9)	
**Educational Achievement**				
Tertiary	7.3 (4.1, 8.7)	6.0 (4.1, 8.7)	6.7 (4.7, 9.4)	0.23
Secondary/Vocational	33.8 (28.0, 40.2)	39.1 (43.9, 43.4)	36.5 (32.2, 40.9)	
Primary	58.8 (52.1, 65.2)	54.9 (50.0, 59.7)	56.9 (51.9, 61.7)	

+ - No meaningful CIs as these age groups were used to create sampling weights.

* P<0.05.

**Figure 1:**
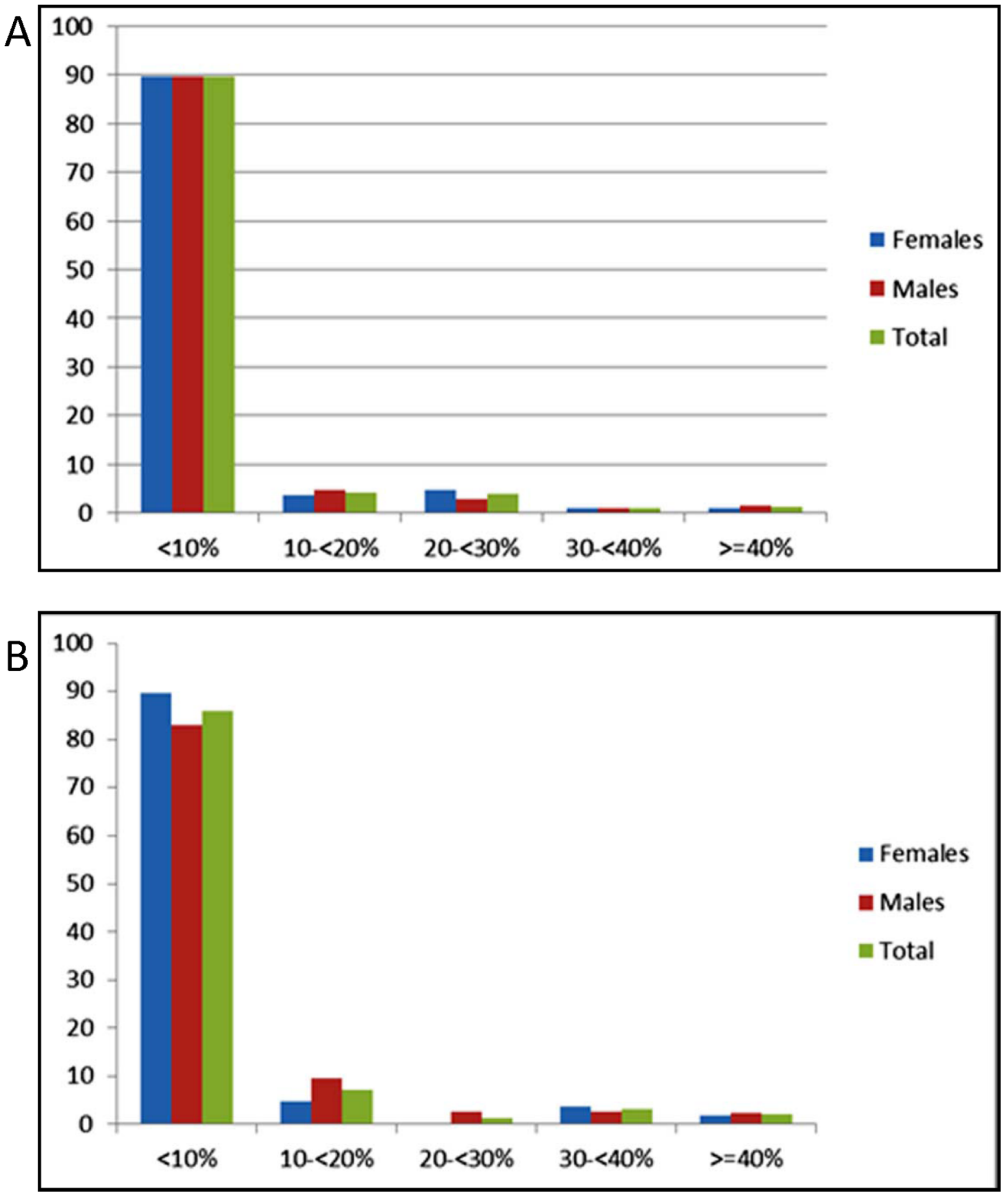
Distribution of 10 year WHO/ISH Cardiovascular Risk Score Categories by Sex, calculated with (A) and without (B) using the cholesterol measurement.

Table 2 shows the distribution of total 10 year CV risk prediction scores in men and women. When the CVD risk was determined for the population without the cholesterol values, the majority (86%) of the sample were classified as being in the lowest (<10%) cardiovascular risk category (Figure 1). The overall CVD risk profile of the population improved marginally after including cholesterol measurement in the estimation of 10 year risk with the proportion at low risk (<10%) increasing to 89.3%. A small proportion of the population was classified as high risk (10 year CVD risk ≥30%). This was approximately 2% (95% CI = 1.4–3.7) or over 14,000 persons if cholesterol was used and 5% (95% CI = 4.0–7.0) or approximately 36,000 if cholesterol measurements were not used. If we include those 40 years and older with self-reported heart attack or stroke then the respective estimates for those at high risk are 5.2%; 95% CI = 4.0–6.9 (using cholesterol in the risk score) and 7.9%; 95% CI = 6.3–9.8 (without using cholesterol in the risk score).

**Table 2:** Population and sex-specific projected 10 year WHO/ISH cardiovascular risk category* distribution (percentages with 95%CI) by age group.

	WHO/ISH Cardiovascular Risk Score Category					
	<10%		10–<20%	20–<30%	30–<40%	≥40%
**Men**						
40–49yrs		97.9(90.6, 99.5)	0.0	0.0	1.7(0.3,10.5)	0.5(0.06, 3.4)
50–59yrs		91.0(84.8, 94.8)	4.5(1.9, 10.2)	4.6(1.8, 11.0)	0.0	0.0
60–69yrs		81.2(71.4, 88.2)	6.6(2.9, 14.2)	7.2(3.4, 14.3)	0.0	5.1(1.6, 15.3)
≥70yrs		58.7(41.0, 74.4)	28.1(15.2, 45.9)	4.9(1.0, 21.0)	3.4(0.5,21.2)	4.9(1.6, 14.0)
All men		89.4(86.1, 92.5)	4.7(2.9, 7.5)	3.0 (1.7, 5.3)	1.0 (0.2,4.4)	1.5(0.7, 3.3)
**Women**						
40–49yrs	96.4(93.8, 97.9)		1.0(0.3, 3.2)	1.7(0.9, 3.2)	0.7(0.2,2.2)	0.3(0.04, 2.2)
50–59yrs	92.2(87.5, 95.3)		2.3(1.0, 5.0)	3.9(1.7, 8.7)	1.4(0.6,3.3)	0.3(0.04, 2.0)
60–69yrs	82.4(74.9, 88.0)		3.7(1.8, 7.1)	9.4(5.4, 15.8)	0.9(0.1,6.5)	3.6(1.6, 8.3)
≥70yrs	61.8(48.8, 73.3)		23.2(15.5, 33.3)	12.7(6.9, 22.2)	0.0	2.3(0.5, 9.6)
All women	89.5(87.3, 91.5)		3.8(2.8, 5.0)	4.7(3.4, 6.5)	0.9(0.4,1.7)	1.1(0.6, 2.1)
**Total (Men and Women)**						
40–49yrs	97.0(94.3, 98.4)		0.5(0.2, 1.6)	0.8(0.4, 1.6)	1.3(0.4,4.4)	0.4(0.09, 1.6)
50–59yrs	91.3(88.0, 93.7)		3.9(2.0, 7.3)	4.1(2.2, 7.3)	0.6(0.3,1.5)	0.1(0.02, 1.0)
60–69yrs	81.3(75.2, 86.2)		5.5(3.2, 9.3)	8.6(5.5, 13.1)	0.5(0.06,3.3)	4.1(1.7, 9.6)
≥70yrs	59.1(48.6, 68.7)		26.5(19.1, 35.5)	9.1(5.4, 14.9)	1.5(0.2,10.3)	3.9(1.7, 8.8)
Total	89.6(87.6, 91.3)		4.3(3.1, 5.7)	3.9(3.0, 4.9)	1.0 (0.4,2.2)	1.3 (0.7, 2.4)

* WHO/ISH calculations performed using total cholesterol.

There was good agreement in participants’ 10 year CVD risk regardless of whether cholesterol was used for prediction (Kappa statistic 0.61 for total population, 0.59 for men and 0.62 for women). We therefore present the remaining results based on 10 year risk estimates that utilized the cholesterol measurement.

Despite the differences in the distribution of cardiovascular risk factors, there was no significant sex difference in the distribution of 10 year CVD risk. As expected, the 10 year risk of a cardiovascular event increased with age, with only 59% of men and 62% of women over 70 years old having a predicted 10 year incidence of cardiovascular disease of <10% (Table 2).

Jamaican women were more obese and less active than Jamaican men. A larger proportion of women also tend to be classified as having low socio-economic status based on education and occupation. As the WHO/ISH risk calculation does not take obesity, physical activity or social status into consideration when estimating overall risk, we examined whether these factors were important contributors to an adverse CVD risk profile, particularly in women. We found no difference in the estimated 10 year CVD risk for men and women according to their BMI and physical activity categories (Table 3). However, sex modified the effect of socioeconomic status on the overall cardiovascular risk. Women in the lower socio-economic categories, based on education and occupation, had a worse cardiovascular risk profile than those in the higher socio-economic categories (See Table 4). This effect was not seen in the men.

**Table 3:** Sex-specific distribution of projected WHO/ISH 10 year risk category* (percentages with 95% confidence intervals) by body mass index and reported physical activity category.

	Risk Score				
	<10%	10–<20%	20–<30%	30–<40%	≥40%
**Men**					
**BMI (kg/m^2^)**					
Normal (<25.0)	90.1 (86.0, 93.2)	4.8 (2.7, 8.4)	2.2 (0.9, 4.9)	0.6 (0.0, 3.8)	2.4 (0.9, 6.3)
Overweight (25–29.9)	88.0 (78.5, 93.7)	5.6 (2.5, 12.1)	3.8 (1.5, 9.3)	2.2 (0.4, 12.0)	0.4 (0.0, 2.8)
Obese (≥30.0)	91.3 (80.2, 96.4)	2.8 (0.6, 11.6)	4.4 (1.3, 14.2)	0	1.5 (0.2, 9.9)
**Physical Activity**					
High	91.0 (85.1, 94.7)	4.2 (2.2, 7.7)	2.6 (0.9, 7.6)	1.5 (0.2, 9.7)	0.7 (0.0, 4.4)
Moderate	92.3(84.4, 96.4)	3.4 (1.2, 9.2)	3.4 (1.2, 9.2)	0	0.9 (0.0, 6.4)
Low	88.6 (78.7, 94.2)	5.8 (2.2, 14.6)	5.6 (2.2, 14.6)	0	0
Inactive	83.5 (72.7, 90.6)	7.2 (2.9, 16.9)	2.2 (0.5, 8.4)	1.5 (0.0, 10.2)	5.6 (2.1, 13.8)
**Women**					
**BMI (kg/m^2^)**					
Normal (<25.0)	89.6 (84.6, 93.1)	3.0 (1.3, 6.5)	5.4 (2.8, 10.0)	0.6 (0.0, 2.4)	1.5 (0.5, 4.6)
Overweight (25–29.9)	90.1 (86.3, 93.0)	4.6 (2.6, 7.7)	3.3 (1.7, 6.1)	0.6 (0.1, 3.1)	1.5 (0.5, 4.0)
Obese(≥30.0)	89.0 (87.2, 91.4)	3.7 (2.8, 5.0)	4.8 (34, 6.6)	0.9 (0.4, 1.7)	1.1 (0.2, 2.3)
**Physical Activity**					
High	93.1 (88.2, 96.1)	1.5 (0.5, 4.5)	4.6 (2.4, 8.9)	0.8 (0.2, 3.6)	0
Moderate	95.9 (92.6, 97.8)	1.9 (0.8, 4.6)	1.6 (0.6, 3.9)	0	0.6 (0.0, 4.0)
Low	89.9 (83.6, 94.1)	3.3 (1.5, 8.8)	4.5 (2.2, 8.9)	0.3 (0.0, 2.5)	1.6 (0.7, 3.8)
Inactive	84.2 (87.2, 91.4)	3.8 (2.9, 5.0)	4.7 (3.4, 6.5)	1.6 (0.7, 2.6)	1.1 (0.6, 2.1)

* WHO/ISH calculations performed using total cholesterol.

**Table 4:** Sex-specific distribution of projected WHO/ISH 10 year risk category (percentages with 95% confidence intervals) by socioeconomic status based on occupation and education.

	Risk Score				
	<10%	10–<20%	20–<30%	30–<40%	≥40%
**Men**					
**Occupation**					
Highly Skilled	94.0 (85.9, 97.6)	4.5 (1.5, 12.9)	1.1 (0.8, 4.1)	0	0.4 (0.0, 3.6)
Skilled	88.3 (82.3, 92.1)	5.4 (3.1, 9.6)	4.0 (1.8, 8.7)	1.5 (0.2, 9.0)	1.0 (0.3, 4.0)
Unskilled	93.5 (74.9, 98.6)	2.3 (0.3, 15.8)	0	0	4.1 (0.6, 25.1)
Unemployed	0	0	0	0	0
Other	76.2 (53.9, 89.8)	10. 5(3.8, 26.0)	10.1 (2.3, 3.5)	0	3.2 (0.4, 20.3)
**Education**					
Tertiary	94.3 (78.8, 98.6)	0	5.6 (1.4, 21.2)	0	0
Secondary/Vocational	94.6 (91.3, 97.9)	2.6 (1.0, 6.6)	0.5 (0, 4.1)	0	1.2 (0.2, 7.7)
Primary	85.9 (80.3, 90.0)	6.5 (3.8, 10.7)	4.1 (2.1, 7.8)	1.7 (0.4, 7.0)	1.9 (0.8, 4.5)
**Women**					
**Occupation** *					
Highly Skilled	93.6 (90.6, 95.6)	1.6 (0.1, 3.7)	3.3 (2.0, 5.2)	0.5 (0.2,1.4)	1.0 (0.3, 3.2)
Skilled	89.5 (83.1, 93.6)	3.7 (1.5, 8.5)	5.6 (2.6, 11.4)	0	1.3 (0.2, 8.2)
Unskilled	89.4 (82.1, 93.9)	3.1 (1.2, 7.9)	5.3 (2.2, 12.3)	1.5 (0.3, 6.5)	0.7 (0, 4.5)
Unemployed	51.6 (13.3, 88.1)	13.5 (1.6, 59.5)	34.9 (6.7, 83.0)	0	0
Other	82.4 (74.2, 88.5)	7.7 (4.3,13.4)	8.3 (4.3, 15.4)	0	1.7 (0.5, 4.8)
**Education** *					
Tertiary	95.6 (87.2, 91.5)	0	4.5 (3.4, 6.5)	0	0
Secondary/Vocational	95.1 (92.0, 97.0)	1.5(0.6, 3.9)	2.1 (1.0, 4.2)	0.8 (0.2, 2.3)	0.6 (0.2, 2.2)
Primary	84.9 (81.3, 88.0)	5.8 (4.2, 8.0)	6.6 (4.6, 9.4)	1.0 (0.4, 2.4)	1.6 (0.8, 3.4)

* P<0.01, WHO/ISH calculations performed using total cholesterol.

## Discussion

Despite the high prevalence of CVD risk factors, the majority of the Jamaicans 40 years and older without established cardiovascular disease have a low (<10%) 10-year risk of a cardiovascular event, regardless of whether total cholesterol was used to estimate the risk. This however still represents a significant number of additional cases of CVD that the country may not be fully prepared for. The sex differences in cardiovascular risk factor burden did not change cardiovascular risk. As expected the 10 year risk of cardiovascular disease events increased with age but did not vary with reported physical activity or obesity status. Women in the lower socioeconomic category, measured using education and occupation, had a more adverse cardiovascular risk profile than those in the higher socioeconomic category.

Our data on the cardiovascular risk factor distribution by sex is consistent with other data from the Caribbean and developing countries undergoing the demographic transition. A STEPS survey of three eastern Caribbean countries reported that women had a higher prevalence of hypertension (35% vs. 21%), diabetes (17% vs. 7%) and obesity (42% vs. 17%), however men were more likely to smoke [25]. In a small cross sectional study from Tanzania, women were more likely to be obese (35% vs. 13%) and were 3 times more likely to have the metabolic syndrome than men [26]. Similar findings were noted in a study of urban Kenyan adults where women were more likely to have a high waist circumference and also had a higher prevalence of the metabolic syndrome [27]. In the Seychelles however, apart from sex differences in smoking prevalence (31% in men and 4% in women) and hypercholesterolemia (25% in men and 32% in women) there were no differences in the prevalence of the other risk factors used for the WHO/ISH CVD risk score [28].

The WHO/ISH 10 year CVD risk distribution using cholesterol measurement from the Jamaican data was similar to that reported in Cuba, Nigeria and the Seychelles where approximately 1% to 2.4% of the population had a risk score of 30% or more [29]. While not specifically reported, there did not appear to be any significant differences by sex, in the proportion within the high CVD risk category (>30% risk) in either Cuba or Nigeria (1.3% of men and 1.0% of women in Cuba and 2.8% of men and 2.1% of women in Nigeria) [29]. In contrast more men in the Seychelles had a higher 10 year WHO/ISH CVD risk score of ≥30% (4.2% vs 0.7%), consistent with their higher CVD risk factor prevalence [28]. While Cuba shares some geographic similarities with Jamaica it has a lower Black population. Nigeria and the Seychelles, while predominantly black, may be at an earlier stage in the epidemiologic transition than Jamaica and many other Caribbean islands.

We explored the effect of obesity on CVD risk as there is a much higher burden of obesity among Jamaican women [9]. In the population surveyed, obesity was not a significant determinant of CVD risk. The lack of an association between CVD risk and body mass index may be due to the effect of obesity being mediated through the intermediate cardiovascular risk factors (hypertension, diabetes and hypercholesterolemia) included as components of the risk score. As a result, despite being an important CVD risk factor, obesity has been omitted from most total CVD risk calculators [30]. This may also explain the absence of an association between reported physical activity and CVD risk scores in our study.

Socioeconomic status in women, but not men, was an important determinant of CVD risk not fully accounted for by the WHO/ISH score. While a more adverse cardiovascular risk distribution was also seen in men in the lower socioeconomic risk categories this effect was not statistically significant. Social inequity has been associated with CVD. Persons in the lower socioeconomic status categories may have higher levels of chronic stress, greater difficulty in accessing care, affording medications and medication adherence [31]. The sex difference in the effect of socioeconomic status on cardiovascular risk is of particular interest and should be explored in future studies.

The WHO/ISH prediction charts identify those at highest risk for a cardiovascular event – which would include both coronary disease and stroke. There are few data from the Caribbean documenting the incidence of each condition and we were unable to identify any studies where a combined incidence of both conditions was reported. Data from hospital based surveillance in Cuba between 2007 & 2008 demonstrated a higher incidence and mortality from cardiovascular disease in men [32] while the Barbados National Stroke Registry showed a higher incidence of stroke in women [11].

Mortality statistics in Jamaica would that suggest sex differences in stroke and coronary disease might account for the absence of a difference in incident CVD. The 2008 mortality statistics found a similar crude coronary disease mortality rate in men and women −35.9/100,000 and 36.7/100,000 but a higher crude mortality from stroke in women (83.3/100,000 vs. 65.9/100,000) [5]. The limitations of routinely collected data combined with sex differences in the diagnosis of coronary disease in women may affect the validity of these data.

The findings of our study are also supported by data from a 30 year follow up (1975–2005) of a black cohort from rural Tobago [33]. In this study cardiovascular deaths were ascertained through a combination of review of death certification data and verbal autopsy interviews of family members and the doctor. Cardiovascular deaths accounted for 38% of male deaths and 55% of female deaths in the 20–59 year age group and 44% of male death and 59% of female deaths in those 60 years and older at enrolment. Sex differences in cardiovascular mortality were significant only in those who were over 60 years old at enrolment, with no sex differences in cardiovascular mortality at younger ages.

The risk score estimates that included cholesterol resulted in fewer persons classified as high risk, while excluding cholesterol from the risk score calculation resulted in a doubling of the proportion at high risk (2% vs 5%). While total cholesterol measurements can be readily obtained in Jamaica, there is limited harm from commencing more aggressive cardiovascular risk prevention efforts in persons who may not be at highest risk. At the current time we are unable to determine which approach to risk assessment is superior and so the decision on which tables should be utilized may have to be determined by the costs associated with each approach and how easily each approach can be incorporated into current clinical practice.

If the total CVD risk approach helps to better identify those persons who will achieve maximal benefit from treatment, this should allow the health system to focus its efforts on the subset of the population in need of special attention to mitigate their short-term cardiovascular risk. Our findings do not suggest that CVD risk reduction campaigns focus more on women than men despite the differences in CVD risk factor distribution. However the higher CVD risk score in women of low socioeconomic status would suggest that special emphasis is placed on this group. Ensuring their access to available social programmes and amenities to facilitate care is particularly important. Formularies for medications that address these risk factors, adequate staffing of government supported health care facilities, flexible clinic opening hours, the provision of transportation, child care and other incentives should be utilized to ensure this population has access to care.

In this study data from a recently conducted, nationally representative, predominantly black population undergoing the epidemiological transition were used to estimate 10 year cardiovascular risk. Limited data are available for these populations. With the exception of Cuba we are unaware of this type of analysis being available for Caribbean countries. Our study collected data on total cholesterol allowing us to explore the effect of cholesterol measurement on overall cardiovascular disease risk assessment. The collection of biological measurements using standard techniques also allowed participants to be better classified according to their overall risk. We were able to exclude persons with established coronary disease from the analysis – for whom the risk estimates are not applicable – to provide better estimates for the population at risk. The absence of a sex difference in 10 year disease risk is counter to many of the assumptions about cardiovascular risk and emphasizes the importance of a better understanding of the pathophysiology of cardiovascular disease in women. While men and women share similar risk factors, differences in the impact of these risk factors on cardiovascular disease have been described [34], [35]. It is possible that being pre-menopausal confers a greater than expected protective effect in women and counters the effects of other cardiovascular risk factors [35]. Additionally sex differences in endothelial function and microvascular disease may explain these differences [35]. These data demonstrate the need for more basic science and epidemiological studies to improve our understanding of the aetiology of cardiovascular disease in women, particularly black women in developing countries, so that those at highest risk can receive benefit from prevention, detection and treatment efforts.

The study however had some limitations. The final sample had an excess number of women and less representation of some age groups as would be expected from the population statistics, suggestive of a frame error. Post stratification weights were used to compensate for this. We studied persons up to age 74 years old and have not included older persons who would be expected to have an even higher 10 year WHO/ISH CVD risk score. As a result our findings may underestimate the risk for the entire population. While CVD events in persons over 75 years old may not be a significant contributor to potential years of life lost, events in this demographic place added demands on the health care system as they form a significant proportion of those admitted to hospital for CVD events or who live with CVD morbidity. Additionally we were unable to explore the effect of other approaches to risk calculation which require additional components of the fasting lipid profile (HDL-cholesterol and LDL-cholesterol measurement) or hsCRP. Due to the lack of cardiovascular incidence data in our population we were unable to assess the validity of the WHO 10 year CVD risk score or compare it with other risk score tools. We were unable to identify any studies that have validated the WHO risk score or compare it with any of the standard risk score calculators.

Despite having a higher burden of CVD risk factors in women we found that there was no sex difference in 10 year CVD risk and a very low proportion of the population at very high risk of a CVD event.

## Supporting Information

Appendix S1 **Sampling methodology and calculation of the survey weighting for the Jamaica Health and Lifestyle II study.**
(DOC)
