# Supplementary material for: Excess Cardiovascular Risk Burden in Jamaican Women Does Not Influence Predicted 10-Year CVD Risk Profiles of Jamaica Adults: An Analysis of the 2007/08 Jamaica Health and Lifestyle Survey
Source: PLoS One. 2013 Jun 21;8(6):e66625. doi: 10.1371/journal.pone.0066625 (PMC3689813; doi:10.1371/journal.pone.0066625)
Supplement: Appendix S1 — Sampling methodology and calculation of the survey weighting for the Jamaica Health and Lifestyle II study. (DOC) [file pone.0066625.s001.doc]

**Appendix S1 - Sampling methodology and calculation of the survey weighting for the Jamaica Health and Lifestyle II study.**

***Sampling Methodology***

Jamaica is divided into fourteen parishes and these parishes are further divided into enumeration districts (EDs) by the Statistical Institute of Jamaica (STATIN). An ED is defined as a geographical area to be enumerated by a single enumerator and consists of up to four hundred dwellings. From the 2006 Labour Force Survey the island was divided into 254 sampling regions (SR). Within SRs, primary sampling units (PSUs) were created from one or more than one contiguous EDs to yield areas and populations of sufficient size to act as clusters for sampling (minimum 80 dwellings). Two PSUs are randomly selected from each SR, yielding a nationally representative sample of 508 PSUs (~10% of EDs nationally). We elected to recruit 30 participants per PSU and with a required sample size of 2914 participants we would therefore need 97 (2914/30) PSUs (clusters).

The number of PSUs per parish was determined by probability proportionate to size (PPS) of the parish and this was achieved by applying the sampling fraction of 97/508 to the number of PSUs within the sampling frame for that parish. EDs were selected and provided by STATIN for our sample selection. Using maps, households were systematically selected beginning at a random starting point and attempts were made to recruit one participant at intervals determined by the size of the PSU. Within each household, a single individual was chosen to participate.

The participant from each household was selected by the KISH methodology. For this method the sampling frame consists of a list of all persons between 15 and 74 years old who spend at least 3 nights a week at that address. A listing of these persons was then created, starting with the oldest eligible member of the household, and the number of eligible participants and the last digit of the questionnaire were used to identify the individual who would be selected for the survey. Interviewers were required to revisit households where adults were not at home at the time of first contact with the household. A minimum of three visits was made before the household/participant is deemed a refusal. The refusal rate was less than 1%.

***Calculation of Survey Weights***

The initial weighting was done to take the probability of selection into account at the level of the primary sampling unit, parish, household and individual within each household.

We created a variable comprising the number of PSUs selected from each parish and used in the study and estimated the average number of individuals living in a PSU in Jamaica based on the number of individuals divided by parish size. From this we were able to obtain the probability of selecting a PSU as follows:

$$\frac{No. of PSUs in parish in sample\times average PSU size}{parish size based on number of 15-74 yr-olds.}$$

Using data from STATIN, there is a minimum of 100 households per PSU. Since the number of persons in the sample in each PSU indicated the number of households selected from each PSU we obtained the probability of selecting each household as follows:

$$\frac{No. of households (persons) in parish in sample}{100}$$

Thus we were able to obtain the raw sampling design weights as follows:

$$\frac{1}{\Pr obability of selecting PSU}\times\frac{1}{\Pr obability of selecting household}$$

We used post-stratification ***(Stata Survey Data Reference Manual, Release 10, Statcorp LP, College Station, Texas, 2007)*** to adjust the sampling weights in order to account for any under-representation of five-year age group by sex categories in the data set which were indicative of a frame error. The post-stratum population sizes according to 2001 census data were also inserted into the data set as a variable.
